# Pregnant Users’ Perceptions of the Birth Plan Interface in the “My Prenatal Care” App: Observational Validation Study

**DOI:** 10.2196/11374

**Published:** 2019-03-28

**Authors:** Juliana Moraes Carrilho, Isaias José Ramos Oliveira, Dimitri Santos, Gabriel Costa Osanan, Ricardo João Cruz-Correia, Zilma Silveira Nogueira Reis

**Affiliations:** 1 Informatics Center in Health Universidade Federal de Minas Gerais Faculty of Medicine Belo Horizonte Brazil; 2 Obstetrics and Gynecology Department Universidade Federal de Minas Gerais Faculty of Medicine Belo Horizonte Brazil; 3 Center for Health Technology and Services Research Universidade do Porto Porto Portugal

**Keywords:** birth plan, perinatal care, usability, mobile health, mobile app, pregnancy, prenatal care, mobile phone

## Abstract

**Background:**

Birth plans are meant to be a declaration of the expectations and preferences of pregnant woman regarding childbirth. The My Prenatal Care app engages pregnant women in an educational intervention for a healthy pregnancy. We hypothesized that users’ positive perception of an in-app birth plan is a relevant step for establishing direct communication between pregnant women and the health care team, based on an online report available on the app.

**Objective:**

This study aimed to evaluate pregnant women’s perception about the communicability of birth-plan preparation using a mobile app.

**Methods:**

This was an observational, exploratory, descriptive study. The methodology was user centered, and both qualitative and quantitative approaches were employed. The tools of the communicability evaluation method were applied. Overall, 11 pregnant women evaluated their experience of using a birth-plan prototype interface. The evaluation was performed in a controlled environment, with authorized video recording. There were 8 task-oriented interactions proposed to evaluate interface communicability with users when using the Birth Plan menu. For evaluating perceptions and experiences, a survey with structured and open-ended questions in addition to the free expression of participants was conducted. The primary outcomes assessed were interface communicability and user’s perception of the Birth Plan prototype interface in the My Prenatal Care mobile app. Secondarily, we involved users in the prototyping phase of the interface to identify bottlenecks for making improvements in the app.

**Results:**

Regarding users’ performance in accomplishing previously prepared tasks, we found that 10 of 11 (91%) women were capable of completing at least 6 of 8 (75%) tasks. A positive relationship was found between the number of communicability problems and the success of completing the tasks. An analysis of the records revealed three communicability breakdowns related to the data entry, save, and scrollbar functions. The participants freely expressed suggestions for improvements such as for the save function and the process of sharing the birth-plan form upon completion.

**Conclusions:**

Users had a positive perception of the Birth Plan menu of the My Prenatal Care app. This user-centered validation enabled the identification of solutions for problems, resulting in improvements in the app.

## Introduction

A birth plan is a description of a pregnant woman’s expectations and preferences regarding childbirth [1,2]. Planning the birth during the antenatal period promotes health education and fosters communication between women and health professionals [3]. The World Health Organization recommends birth plans as a part of prenatal care [4]. The wide use of such planning can mitigate excessive medicalization during childbirth and empower women to be the protagonist during childbirth [5]. Pregnant women should receive comprehensive health care that is continuous and customized from the prenatal period until delivery [6]. Hence, relevant clinical information needs to be available at the time of the delivery, including a birth plan.

Mobile apps related to pregnancy, birth, and childcare are important information sources for users, as they combine education and functionalities of communication to support the self-management of health [7]. App adherence can influence pregnant women’s behavior such as keeping prenatal care schedules, improve health care, and promote self-care [8].

**Figure 1:**
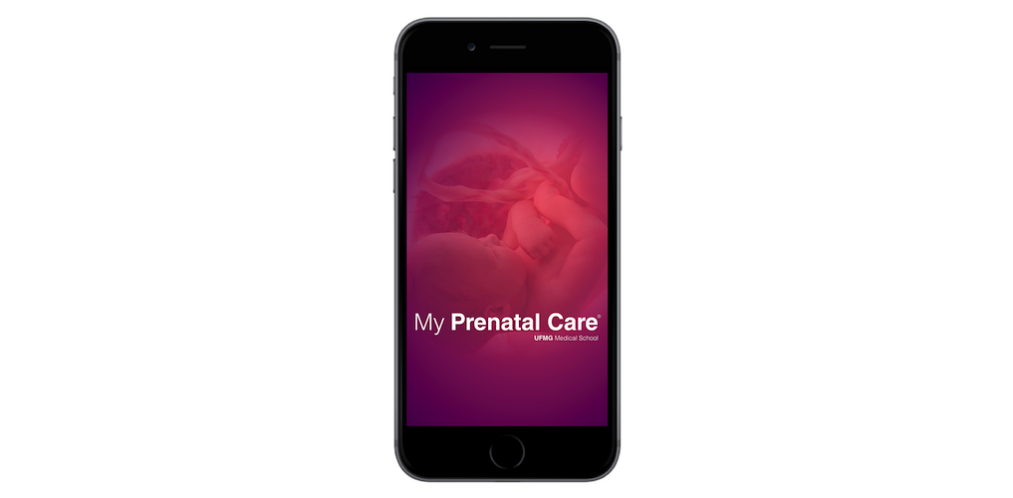
The My Prenatal Care app.

**Figure 2:**
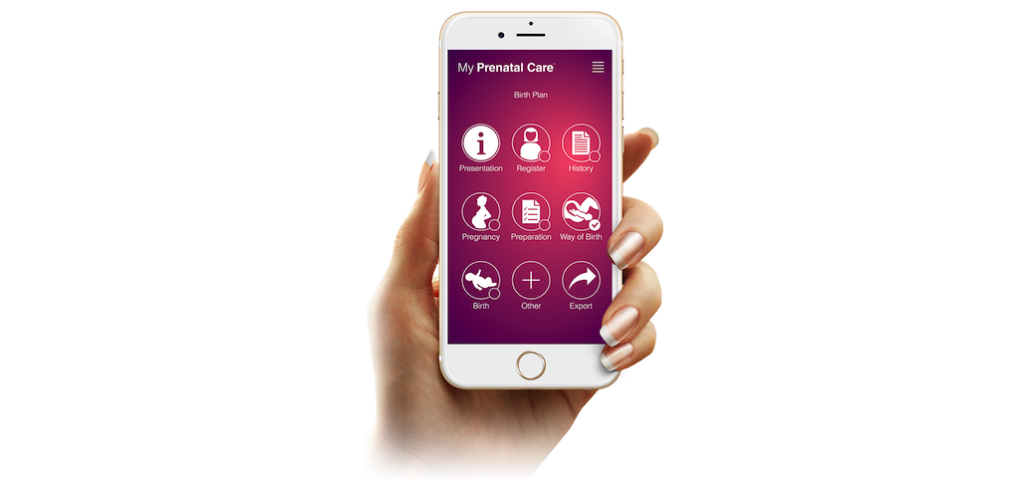
The Birth Plan menu within the main menu of the app.

The My Prenatal Care app (Figure 1) was created in 2016 to engage pregnant women in an educational intervention for a healthy pregnancy, delivery, and puerperium [9]. This app is part of a project that aims to highlight the importance of gestational dating at birth to recognize premature newborns. Developed by an academic and multidisciplinary team of researchers, this app is organized into the following three sections: My Pregnancy, The Delivery, and My Baby Is Born. Domain specialists have validated the scientific content in the app, which is offered in Portuguese, English, and Spanish [9]. The app is available for free in app stores, with 100,930 downloads reported until November 4, 2018, of which 90,290 downloads are from Android devices and 10,640 are from iOS devices. Before introducing the birth plan in this app, an exploratory study analyzed a planning proposal to support obstetric care and the development of an interoperable open electronic health record (EHR) [10] standard for entries of clinical information in electronic system interfacing [11]. The proposed Birth Plan menu contains expectations for the birth moment and parts of individual medical history, which can be declared by the users at any point in time. The interface menu was prepared with the following eight sections: Identification, My History, My Pregnancy, Preparations, My Childbirth, Delivery, Other desires and expectations, and Share (Figure 2) [11].

The human-centered design and development of systems require that apps be made usable and useful by focusing on the users [12]. Usable systems are beneficial for supporting an appropriate human-system interaction and fostering patient adherence [13,14]. In this context, communicability is an attribute of software that effectively conveys to users the underlying design intent and interactive principles [15]. The communicability evaluation method is an approach to evaluate the quality of the designer’s communication with the user through the interface. Proposed by de Souza (2005), this evaluation allows the identification of communication breakdown with the computational artifact during user interaction [16]. We hypothesized that a positive perception of users on the in-app birth plan template is an essential step for establishing direct communication between pregnant women and the health care team at birth, based on an online report available on the app. With the early involvement of users in the prototyping phase of the interface, resolving communicability barriers would result in improvements that promote adherence to and foster the utility of this app. The present study aimed to validate an interface for birth-plan preparation in the My Prenatal Care app by examining interface communicability and the perceptions and experiences of a sample of the target population.

## Methods

### Study Design

This observational, exploratory, descriptive study employed an interdisciplinary approach combining health and information sciences. The methodology was user centered and employed qualitative and quantitative approaches to assess pregnant women’s experience of using the Birth Plan menu of the My Prenatal Care mobile app. Pregnant woman who agreed to participate in the study individually answered a semistructured questionnaire (Multimedia Appendix 1). Their responses were analyzed to identify their sociodemographic and obstetric profiles and assess their previous experience with mobile technology.

To identify the points of communication breakdown, task-oriented interactions were analyzed by two of the three steps in the communicability evaluation method—tagging and interpretation. Communication breakdowns were defined as disruptions occurring during user interaction based on the computational artifact acquired from video analysis, according to a prespecified set of utterances [15]. Tagging involves selecting words from utterances regarding a user’s reaction during interaction [16]. Communication was assessed based on the ruptures identified in the communicability evaluation method [15,16]. Finally, users’ experiences were examined by conducting an individual survey. The study was conducted in Portuguese. In this report, we intended to achieve comprehensiveness and quality with respect to the effectiveness of digital programs proposed in the mHealth Evidence Reporting and Assessment checklist published by the World Health Organization [17].

### Recruitment and Settings

The research protocol was approved by the Ethics Committee of the Universidade Federal de Minas Gerais, with the national register number CAAE-68076617.2.0000.514 in Plataforma Brasil. All participants were informed that participation was voluntary and were provided the details of the study. To ensure voluntary participation, they were allowed to withdraw their consent at any time, without any consequences. Written informed consent was obtained from each participant. To avoid influencing the users’ perceptions positively, they were not provided any direct or indirect gratification. Additionally, there was no burden on the participants, except their time.

The participants were recruited from the university’s prenatal care center. High-risk pregnancies are often referred to this public and teaching health care institution. Data were collected from December 2017 to January 2018. The participants attended institutional focus groups led by obstetric nurses before enrollment in the research. During these group sessions, the importance and objectives of elaborating a birth plan and preparation for birth were discussed. This educational approach is routine in this unit and is not part of the methodology.

### User Sampling

Considering the significant variance in the computer skills of our population, this study used a convenience sample of 11 participants to evaluate users’ perceptions of the birth-plan interface tested in this study. Based on a previous report, the detection of usability problems requires at least five users, because the function of the number of users tested or the number of heuristic evaluators is modeled as a Poisson distribution [18]. According to a study by Nielsen in 2000, 85% of usability problems can be revealed by involving five users during iterative design evaluation [19]. However, for a comprehensive analysis of problems, the number of required users is almost doubled [20].

A total of 15 women expressed interest in participating in this study during their prenatal care visit. Three women dropped out of the study citing fatigue during the ethics information sessions before communicability testing. One pregnant woman was excluded during the tests because of visual impairment. The final sample comprised 11 participants.

### Study Procedures

#### Pilot Evaluation

We conducted a pilot evaluation to prepare a controlled video-shooting environment. Four women (of whom two were not pregnant) who were not part of the main sample were invited to validate the setting, equipment, and instruments prepared for the monitored interaction between the participants and the interface. First, the equipment and mobile devices were tested and adjusted for effective recording of the interactions in two nonpregnant women. Second, data-collection instruments to be used for the participants and for evaluation of communicability were tested in two pregnant women. Poor image quality revealed the need to replace one video camera, while the data-collection instruments were considered adequate. We captured images during the test from four different angles using one tablet (a 7-inch Samsung tablet) and two Sony high-definition digital cameras supported by tripods and software. During the execution of the predefined tasks, one angle captured the manipulation of the mobile device, one recorded the face, the third recorded and entire body. The AZ-Screen-Recorder software (Hecorat Global Technology, Hanoi, Vietnam) continuously captured images of the mobile interface during the experiments. Further, observational notes were taken during filming.

#### Communicability Evaluation Method

In order to identify and adjust human-computer interaction problems, we used the communicability evaluation method. The focus was on the quality of the user interaction while using the Birth Plan prototype interface. The researcher verbally and in writing advised the participants about the task to be executed in the app. The participants were free to talk during the test and received a fictitious case report (Multimedia Appendix 2) based on a real situation, in which a pregnant woman named Ana, who had already created a birth plan using the My Prenatal Care app, wanted to change some aspects of the birth plan. The participants were asked to perform eight modifications in the birth plan using a smartphone. The same smartphone with the same internet speed was used by the users in all tests—a Samsung Galaxy J5 smartphone with an Android-based operating system connected with 3G broadband internet. Each task purposefully included one of the menus of the Birth Plan prototype interface for exploring all the menus (Figure 2).

The experimental scenario was exclusively used for testing in order to avoid interruptions and displacement. No help was provided by the researchers or third parties. Identical task-oriented interactions were used for each participant individually. The evaluation started with a fictitious case in which the strategy for the communicability test was implemented by minimizing personal involvement or deep reflections. This procedure allowed the users to work impartially only on the tasks without personal opinions.

Video record analysis, tagging, and interpretation were performed for identifying breakdowns in communicability and problems in human-computer interaction by detailing the moments and interfaces in which they occurred [15,16]. We created a tag for each difficulty expressed by the user based on the following expressions from the metacommunication message approach recommended by de Souza [16]: “What’s this?” “Why doesn’t it?” “Help!” “Where is it?” “What now?” “What happened?” “Oops!” “Where am I?” “I can’t do it this way,” “Thanks, but no, thanks,” “I can do otherwise,” “Looks fine to me,” and “I give up.”

The video recordings and observation notes were reviewed repeatedly for analysis and tagging using the Filmora software (Wondershare Technology, Shenzhen, China). Communication breakdowns were analyzed to measure interface communicability for the users. Two authors (JC and IO) judged the participants’ interaction with the interface. All video material and notes were first analyzed individually and then combined based on consensus between judges, following the communicability evaluation method [16]. Communicability was measured by the frequency of breakdowns in each task and by each tag. The outcomes were classified as follows: tasks completed without interaction breakdown, tasks completed with interaction breakdown, and unfinished or unrealized tasks. The frequency of instances of communicability breakdown in each category was used for subsequent analyses.

#### User Experience

An individual in-person evaluation assessed users’ experience regarding the interaction with the system. We conducted a semistructured survey with one question on the difficulties experienced in completing the test and two open-ended questions allowing for free expression to provide suggestions and record experiences/opinions (Multimedia Appendix 3). Adjectives or expressions associated with the individual experience during the test were captured. This evaluation aimed to identify the difficulties and user perception in using the in-app birth-plan interface.

#### Data Analysis

All data were stored in an Excel database and analyzed in IBM SPSS 22.0 (IBM Corp, Armonk, NY). Descriptive statistics explored the characteristics of the participants, summarized according to the nature of the variables. The numerical variables were described using minimum and maximum values, average, and SD. The variables are presented using absolute and relative frequencies. The responses for the difficulties experienced in performing the tasks were presented in terms of absolute and relative frequencies. The qualitative evaluation described how positive the users’ experience was in interacting with the app.

## Results

### Participant Characteristics

The age of the 11 pregnant women enrolled in this study ranged from 18 to 39 years, with an average age of 30.7 (SD 6.5) years (Table 1). Two participants had a postgraduate level of education. However, most of them had studied only until the high school level (7/11, 64%). Marital status or stable marriage was predominant (9/11, 82%), and eight of the participants (8/11, 73%) were employed.

Regarding the obstetric profile of the participants, gestational age ranged from 17 to 39 weeks of gestation and almost half of the participants were parous (6/11, 55%). However, only half (3/6, 50%) of the parous participants classified their previous childbirth experience as good. None of the participants had previous experience in making a birth plan.

All participants owned a smartphone, and 10 of 11 (10/11, 91%) had access to mobile internet. The same frequency was observed for experience with a device with an Android-based operating system. The duration of daily smartphone use was 3 hours or more for 6 of the 11 women (55%), while 4 of the 11 (37%) used their phones for more than 5 hours per day. WhatsApp was the most frequently used app (9/11, 82%). Seven (7/11, 63%) participants reported no experience with health and well-being apps, while three had used an app to support their present gestation.

### Communicability Evaluation Method

Task 8 was the only task that was completed by all the participants (Table 2). It pertained to sharing a birth plan output file with a person from the participant’s WhatsApp contacts. The participants took the longest time to complete the tasks for the “Identification” menu (Task 1), followed by the “Other desires or expectations” menu (Task 7). The lowest performance was observed for Tasks 1, 4, and 6, among which Tasks 4 and 6 pertained to accessing specific options in the menu and changing them.

The number of tags found in the analysis of the video recordings, after considering the observers’ notes, is presented in Table 3. The distribution of the tags by activity and participants allowed a more in-depth analysis of users’ interaction with the interface, thereby adding new insights and identifying communicability problems.

The most-frequent tag—“What happened?”—was associated with starting the “Save” action 16 times. We observed repeated attempts to use the “Save” action after finishing tasks because the provided feedback was not perceived by the user. The second most–frequent tag, with 14 instances, was “Where is it?” This tag symbolizes the difficulty of the user in finding functionalities in the interface to perform the required actions [16]. There were 10 instances of “Looks fine to me.” In these situations, the user was convinced of having performed the task successfully. However, the users did not actually perform the task successfully. We interpreted this as a problem in human-computer interaction due to an incomplete message from the designer of the interface.

**Table 1:** Characteristics of the study participants (N=11).

Variables		Values
**Demographic characteristics**		
	Age (years), mean (SD), (range)	30.7 (6.5), 18-39
	White race, n (%)	6 (55)
	High school or lower education, n (%)	7 (64)
	Not single, n (%)	9 (82)
	Employed, n (%)	8 (73)
**Obstetric information**		
	Gestational age (weeks), mean (SD); range	32.5 (8.2); 17-39
	Nonparous, n (%)	6 (55)
	Good experience with previous birth^a^, n (%)	3 (50)
	No prior experience with a birth plan, n (%)	11 (100)
**Mobile technology experience, n (%)**		
	Smartphone ownership	11 (100)
	Having access to mobile internet on the smartphone	10 (91)
	Using a smartphone with an Android-based operating system	10 (91)
	Most frequently used app is WhatsApp	9 (82)
	Using a smartphone for ≥ 3 hours daily	6 (55)
	Never used a mobile health app	7 (64)

^a^N=6 for parous women.

**Table 2:** Outcomes for using the Birth Plan prototype interface according to each task completed (N=11).

Task number	Task	Participants who performed the task, n (%)
1	Finding the Identification menu: Enter the phone number 122334455 for the primary care contact	8 (73)
2	Finding the My History menu: Change parity to 2	10 (91)
3	Finding the My Pregnancy menu: Write “I used ferrous sulfate”	10 (91)
4	Finding the Preparations menu: Change the option to “Yes” for Photographer/Movie	8 (73)
5	Finding the Birth Position menu: Change to “squatting position”	10 (91)
6	Finding the Birth menu: Choose the “By myself” option in the baby’s first shower	8 (73)
7	Finding the Other desires and expectations menu: Write “I would like to hire a professional photographer”	10 (91)
8	Finding the Share menu: Share the Birth Plan with Ana’s friend through WhatsApp	11 (100)

**Table 3:** Frequency of tags analyzed with the communicability evaluation method to assess user interactions and the Birth Plan prototype interface.

Tag	Task 1	Task 2	Task 3	Task 4	Task 5	Task 6	Task 7	Task 8	Total
Where is it?	2	1	2	2	2	1	0	4	14
Looks fine to me	2	1	1	2	1	3	0	0	10
What happened?	5	3	2	2	3	1	0	0	16
Help!	3	0	0	1	0	0	0	0	4
Oops!	3	1	0	0	0	0	0	0	4
Where am I?	0	0	0	1	0	0	0	0	1
What now?	1	0	0	0	0	0	0	4	5
I give up	1	0	0	1	0	0	0	0	2
Total	17	6	5	9	6	5	0	8	56

The analysis of the execution of Task 4 presented nine instances of tags. A higher difficulty could be attributed to the positioning of this entry in the menu options. We intentionally asked the participants to localize this entry in the bottom edge of the menu to analyze the interaction between the users and the scrollbar. We noticed a positive relationship between the number of communicability breakdowns and success in completing tasks, except for Task 8, which had eight instances of tags. A breakdown of communication occurred because the users chose the option “Share” in the Birth Plan menu; the symbol that indicated loading lacked text and was not sufficient for users to understand its function. Another point of breakdown was when the users did not immediately realize that Ana’s friend (a fictitious contact for sharing the birth plan) could be one of their contacts.

Task 7 was not assigned any tag, and 91% (10/11) of the users completed the task. Tasks 1 and 4 were associated with the highest number of tags, indicating a higher frequency of communication breakdown compared to the other tasks. These tasks had the lowest completion rate (8/11, 73%). Task 1, which received 17 tags, pertained to the first contact with the Birth Plan prototype interface. Therefore, the initial difficulty experienced by the users was expected. However, we found communication breakdowns in two other instances: One was related to a difficulty in finding the option “Phone Number” in the Birth Plan menu, and the other was related to the feedback of the save function.

Table 4 presents the individual performance of users for each task. Most of the tasks previously prepared to be performed in the Birth Plan menu (6/8, 75%) were completed by the majority of pregnant users (10/11, 91%). Three users (3/11, 27%) completed all tasks despite interaction breakdown. Five users (5/11, 45%) completed the majority (7/8, 88%) of the tasks. The lowest performance was observed in a user with texting difficulty because of a congenital malformation on her fingers (P2).

### User Experience

Nine participants (9/11, 82%) reported having no difficulties when they were preparing a birth plan using the app. Two of them reported impairments associated with their ability to handle smartphones, but the impairments pertained to their ability, in general, and not solely during the study. They expressed spontaneous phrases about their experience of and opinion about the Birth Plan prototype interface (Multimedia Appendix 4). The content of responses that included adjectives and expressions associated with the app or the experiment were identified and summarized according to opinion (Table 5).

**Table 4:** Users’ individual performance on the communicability evaluation method according to breakdown.

Participant number	Tasks completed without breakdown of interaction	Tasks completed with breakdown of interaction	Unfinished or unrealized tasks
1	4, 6, 7, 8	1, 2, 5	3
2	None	1, 2, 3, 8	4, 5, 6, 7
3	2, 3, 4, 5, 7	8	1, 6
4	2, 3, 5, 6, and 7	1, 4, 8	None
5	2, 3, and 7	1, 5, 8	4, 6
6	2, 4, 6, and 7	3, 5, 8	1
7	1, 3, 5, 6, 7, 8	2	4
8	7, 8	1, 2, 3, 4, 5, 6	None
9	3, 4, 5, 6, 7	1, 8	2
10	2, 3, 4, 5, 6, 7, 8	None	1
11	4, 5, 6, 7, 8	1, 2, 3	None

**Table 5:** Tally of users’ opinions regarding the Birth Plan prototype interface.

Users’ opinions		Users
**Positive perceptions**		
	Communication	P5^a^, P9, P11
	I liked it	P5, P6, P7, P8
	Great or excellent	P1, P7
	Good or very good	P2, P4, P10
	Cool	P3
	Interesting	P3, P6, P8, P9
	Relaxed	P10
**Process-related perceptions**		
	Learning	P2, P10
	Improvement of care	P4
	Practicality	P11
	Speed	P11
	Ease	P7
	Monitoring autonomy	P1, P5, P9

^a^P: participant.

## Discussion

### Principal Results

**Figure 3:**
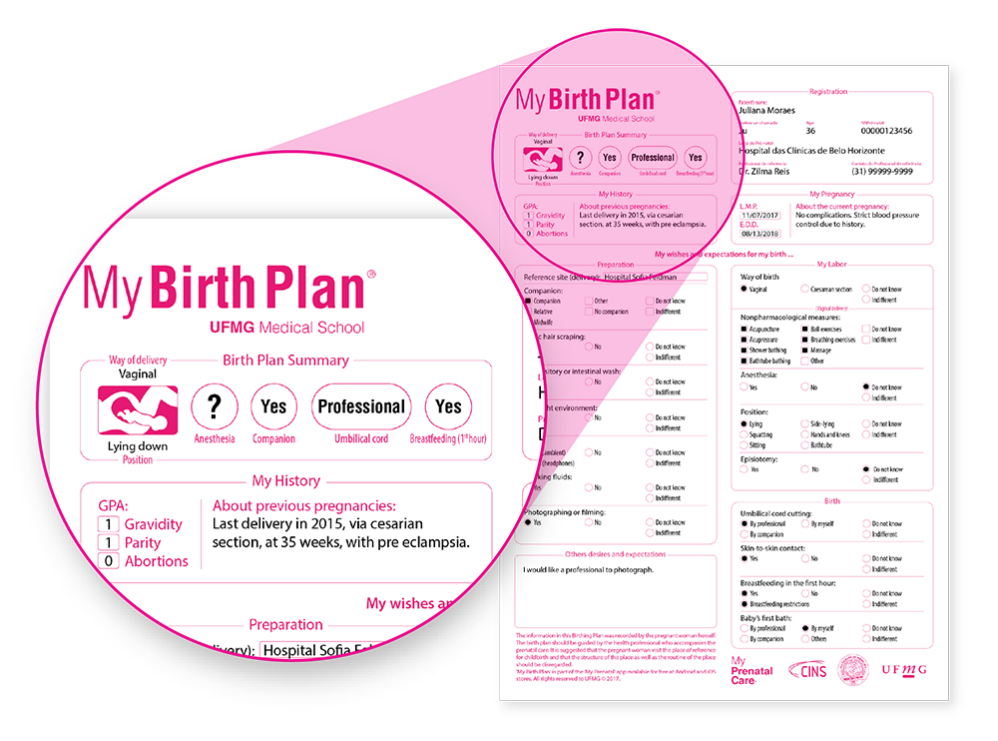
A completed Birth Plan ready to be shared in the My Prenatal Care app. The amplified area shows the structured questions and free descriptions by the pregnant women.

In this study, we validated a birth-plan interface in terms of its communicability based on the interactions and perceptions of pregnant users for creating an in-app birth plan. Our main finding was the positive perception of users regarding birth-plan preparation in the app. An advantage of using a mobile app for creating and sharing birth plans is that it is promptly available for future retrieval anywhere the birth may take place. This document is personal to each woman once she has expressed her wishes and should be reviewed during every follow-up visit and modified, if any complications arise [4]. Planning birth is a part of prenatal care, and the app interface supports flexibility in the user’s answers and any subsequent modifications made before delivery. The offered interface contains a self-explanatory questionnaire with structured and preformatted questions and answers as well as opportunities for free description (Figure 3). Previous reports showed that the provision of skilled care during delivery and women’s satisfaction can be improved with communication between pregnant women and health professionals through prior planning for birth [21]. We hope that during labor, discussing the birth plan with caregivers can be helpful by accessing it on the smartphone or transferring it from the app to local information systems.

To play an active role in identifying a pregnant woman’s needs, birth plans are being introduced during prenatal care by involving women in discussions with future care providers and listening to their desires and flexibilities regarding the birth experience [22]. An editable birth plan as an in-app function should enhance interactions between users and the app when attending to interactive requirements. The lack or low number of instances of communicability breakdown, which were identified using the communicability evaluation method in this study, were related to completed task executions. This methodology, a systematic and qualitative procedure, adequately evaluated users’ experience of interaction with the interface by emphasizing the aspects of communication, as was done in previous studies [15,23]. When applying the communicability evaluation method in this study, we expected that the early involvement of pregnant women users in the prototyping phase of the birth plan interface would help identify bottlenecks and improvements that need to be made in the app. We believe that successfully evaluating human factors when analyzing the human-interface interaction can promote users’ adherence to the electronic birth plan.

With respect to the complete execution of tasks and based on the analysis of the communicability breakdowns identified by the communicability evaluation method, the results pointed out the areas that could be enhanced in the app. Regarding improvements in the interface for solving communication problems, we introduced some modifications. The “Save” message now appears in a new window that opens automatically at the center of the screen, thereby signaling the saving process. Another modification was the appearance of the text message “Loading” when the “Share” command is used. For better interaction with the scrollbar, we plan to use an animation to signal the bar.

Our findings revealed favorable outcomes for the overall user experience. Most of the pregnant users did not report difficulties when performing the tasks with the Birth Plan prototype interface. The user experience survey revealed that all of them expressed good opinions, and “I liked it,” “Interesting,” and “Communication” were some of the positive aspects that the users associated with the app (Multimedia Appendix 4). Impairments in task executions were associated with the participants’ ability to handle smartphones. Moreover, one of the participants had a congenital malformation in her fingers. A challenge for future versions of the app is offering better opportunities to users with disabilities.

Another point to highlight is the sharing of clinical data among systems. Health data include relevant information that might support medical decisions. EHRs demand essential properties such as sharing data with semantic interoperability, preserving flexibility for modifications, and fostering efficacy to promote communication among stakeholders [24]. Credible apps related to perinatal care should be developed and managed in partnership with qualified health care professionals [7]. In this study, we first prepared a standardized model of information to transfer the birth-plan report from prenatal care to childbirth through digital channels [11]. The framework for the birth plan was based on a structured model of information and a reference template based on open EHR specifications [10]. In fact, the interoperability of the clinical data among information systems was not tested. However, because medical concepts and a data format were specified, the entries for the birth-plan questions can be correctly interpreted by a health professional or are understandable in an electronic medical record.

### Limitations

This study had some limitations. Although the sample was considered adequate to validate tests of human-computer interaction, it did not allow for inferential statistical analysis. Regarding penetrability, technologies directed toward pregnancy care require flexibility in order to support different models of birth, specific circumstances, and cultural meanings of childbirth. We can assess the scope and usefulness of this tool only by monitoring the adoption of the app. In the future, we wish to conduct another study involving different centers, cultures, and languages, as the app is currently offered in only three languages. Furthermore, we would like to compare pregnant women’s perceptions when birth plans are declared in a traditional manner such as orally, in writing, or through information technology.

Another limitation was the controlled experimental scenario, which may not reflect real-world situations. However, the present study avoided bias in analysis owing to differences in the speed of the internet connection and smartphone performance.

An important factor is the limitation of the use of technology in a health care setting. During pregnancy, it is advisable that prenatal care be provided by health professionals directly to pregnant women. Any impact of a birth plan in improving the quality of care cannot be attributed only to the app, but also to best obstetric practices [25].

### Comparison with Prior Work

Mobile apps have become a primary source of health guidance for people. Other reports with similar target users have revealed that internet access through websites or mobile apps is useful in helping women adopt a healthy lifestyle during pregnancy and in self-managing their prenatal care [26,27]. In fact, the introduction of an obstetric EHR has improved documentation completeness [28]. The internet may be a promising modality for communication toward the provision of comprehensive health care during pregnancy [29]. However, the quality and safety evaluation of health and well-being apps remain inadequate [30]. The user interface design, performance, and stability of the software program is a part of this validation. The interaction among conventional mobile systems is based on a modified version of a human-desktop computer interface [31]. During the development of personal health records, human-centered design allows the development team to focus on users’ needs to increase the satisfaction with and acceptance of the system [12]. Our project aimed to introduce the birth plan as a personal health record, prepared by pregnant women in the community and assessed by a free and institutional app with a significant number of users.

### Conclusions

The interface for birth plan–preparation tested in this study, which was provided through a mobile app, was perceived positively by pregnant study participants. Its user-centered validation enabled the identification of new solutions to solve communication problems, resulting in improvements to the app. This experience revealed real-world perspectives on the communicability for creating an in-app birth plan and on supporting information sharing among pregnant women and the health care team.

## Appendix

Multimedia Appendix 1 Questionnaire on users’ characterization.

Multimedia Appendix 2 Fictitious case report.

Multimedia Appendix 3 User experience test survey.

Multimedia Appendix 4 Qualitative analysis of the perceptions of users.

## Abbreviations

EHR: electronic health record
P: participant
