# Supplementary material for: Pregnant Users’ Perceptions of the Birth Plan Interface in the “My Prenatal Care” App: Observational Validation Study
Source: JMIR Form Res. 2019 Mar 28;3(1):e11374. doi: 10.2196/11374 (PMC6458531; doi:10.2196/11374)
Supplement: Multimedia Appendix 1 [file formative_v3i1e11374_app1.pdf]

Appendix A

Questionnaire on users' characterization

I - Demographics

1-Identification number: _____
2-Your age (in years): _______
3- Ethnicity self-declaration: () white () brown () black () indigenous () yellow
4-Religion: ______________________________
5-Education degree: ___________________________
6-Origin: ____________________________
7-Gestational age, based on the last menstrual period or ultrasonography (weeks):_______
8-Marital status: () married-cohabitant () single () other_________________
9- Occupation: ________________

II- Health history

1-How many births have you had? ( ) none ( ) 1 ( ) 2 ( ) 3 ( ) 4
2-If you already had, how was your experience? ( ) positive / good ( ) negative / bad ( ) neither positive nor negative
3-Have you performed any Birth Plan before? ( ) Yes ( ) No
3.1- If yes, how was your experience in carrying out the Birth Plan?
____

III- Technology experience

1-Do you own a cell phone? ( ) Yes ( ) No
1.2 – If yes, which its operating system: ( ) IOS ( ) Android
2-Do you have Internet accessible on your cell phone? ( ) yes ( ) no
3-Which application do you most access on your cellphone? ( ) none ( ) Whatsapp ( ) Facebook ( ) Instagram ( ) Games ( ) other: ______________
4-What is your daily cellphone usage time, considering average? ( ) less than 1 h ( ) between 1 and 3 hs ( ) between 3 and 5 h ( ) more than 5 h
5-Have ever used any health-related application? ( ) Yes ( ) No
5.1-iiIf yes, which ones? ____
