# Supplementary material for: Pregnant Users’ Perceptions of the Birth Plan Interface in the “My Prenatal Care” App: Observational Validation Study
Source: JMIR Form Res. 2019 Mar 28;3(1):e11374. doi: 10.2196/11374 (PMC6458531; doi:10.2196/11374)
Supplement: Multimedia Appendix 2 [file formative_v3i1e11374_app2.pdf]

Fictitious case report

Ana Elise is a pregnant woman who the chronology is on 27th weeks of gestation. When she had 13 weeks of gestation, she prepared her birthing plan using the My-Pre-Natal-application. Now, she was invited to participate in the focus group to support the pregnancy, and the birth plan preparation was mentioned. In this group, several doubts were solved, and new ideas came into her mind. She talked to her husband about and to the caregiver who support her during prenatal care, and now she decided to change some issues in the birth plan.

You will help Ana Elise making the changes she would like in the Birth Plan interface, using the My-Prenatal-application. The directions are:

- Please, enter the "Birth Plan" functionality in the application.
- Take a look at the menu options
- Now, make modifications in the Birth Plan, as desired by Ana and save them, as follows:

1- Finding Identification menu: Entry of the phone number 122334455 for the primary care contact
2 - Finding My History menu:<br>Change parity for 2
3- Finding My Pregnancy menu:<br>Write “I used ferrous sulfate”
4- Finding Preparations menu:<br>Change option to “Yes” in in Photographer/Movie
5- Finding Birth Position:<br>Change to “squatting position”
6 - Finding Birth menu:<br>Change for “by myself” option in the baby's first shower
7 - Finding Other wishes menu<br>Write “I would like to hire professional photography
8 - Finding Share menu:<br>Share the Birth Plan with Ana’s friend through WhatsApp
