# Supplementary material for: Pregnant Users’ Perceptions of the Birth Plan Interface in the “My Prenatal Care” App: Observational Validation Study
Source: JMIR Form Res. 2019 Mar 28;3(1):e11374. doi: 10.2196/11374 (PMC6458531; doi:10.2196/11374)
Supplement: Multimedia Appendix 3 [file formative_v3i1e11374_app3.pdf]

User experience test survey

Now, we wanto to know about yor experience during the Birth-Plan preparation an appliation. You can expresse suggestions for improvements

1-What were the issues you had the most difficulty to execution:

() My ability to handle applications in general
() Regarding how the content was exposed in this application
() My ability to handle cell phones in general
() I had no difficulties, considering the way I performed the tasks
( ) Other opinion:_______ ____

2-Do you have any suggestion to improve the Birth Plan in this application? ( ) Yes ( ) No.
If yes, please describe your suggestions: ______________________________________

3- Now, Please describe your experience or opinion when using the Birthing Plan in an application:____

We thank you for your cooperation!
