# Supplementary material for: Pregnant Users’ Perceptions of the Birth Plan Interface in the “My Prenatal Care” App: Observational Validation Study
Source: JMIR Form Res. 2019 Mar 28;3(1):e11374. doi: 10.2196/11374 (PMC6458531; doi:10.2196/11374)
Supplement: Multimedia Appendix 4 [file formative_v3i1e11374_app4.pdf]

Qualitative analysis of the perceptions of users

Spontaneous phrases of the users about their experience and their opinion about the BP-prototype-interface. Original Portuguese' version with the English' translation:

1. "***Ótimo**. A mulher deveria de fato ter **autonomia** em escolher anestesia*"
   "**Great.** The woman should have **autonomy** in choosing anesthesia" (P1)

2. "***Muito bom, aprendi** coisas que não sabia com o aplicativo*"
   "Very good, I **learned** things I did not know with the application" (P2)

3. "*Achei **legal e interessante***"
   " It was **cool and interesting**" (P3)

4. "***Muito bom** este aplicativo, ajuda a **melhorar nossa assistência***"
   "**Very good** this app, it helps to improve our assistance" (P4)

5. "***Gostei** do aplicativo, porque você tem **possibilidade de falar o que deseja***" "I **liked** the application because you have the a**bility to expresse what you want**" (P5)

6. "***Gostei** do aplicativo, **muito interessante***"
   "I **liked** the application, **very interesting**" (P6)

7. "***Gostei, achei ótimo**, vai ser muito **bom para quem utiliza**. Tendo o aplicativo **ajuda** muito, será **excelente**. Muito **fácil de usar***"
   "I **liked it**, I think it's **great**, it's going to be **very good for those who use it**. Having the app **helps a lot**, it will be great. Very **easy to use** "(P7)

8. "*Achei **interessante, gostei***"
   "It was **interesting, I liked it**" (P8)

9. "***Muito interessante**, uma forma de **colocar sua vontade, de se expressar***" "**Very interesting**, a way of putting our desires, **expressing myself**" (P9)

10. "*Bom para gestante **acompanhar**, ficar mais **tranquila** nessa fase que não temos tanto **conhecimento***"
    "Good for pregnant women **to accompany**, to be **calmer** at this stage that we do not have so much **knowledge**" (P10)

11. " ***Rápido, prático**, e sem dúvda, a melhor funcionalidade é **poder compartilhá-lo**. Não adianta a informação ficar só comigo*"
    "**Quick, practical**, and without a doubt, the best functionality is to **be able to** share it. It's no good letting the information only stay with me" (P11)
